# Changes of fat-mass and obesity-associated protein expression in the hippocampus in animal models of high-fat diet-induced obesity and D-galactose-induced aging

**DOI:** 10.1186/s42826-020-00046-0

**Published:** 2020-07-06

**Authors:** Min Soo Kang, Woosuk Kim, Tae Hyeong Kim, Hyo Young Jung, Hyun Jung Kwon, Dae Won Kim, In Koo Hwang, Jung Hoon Choi

**Affiliations:** 1 Department of Anatomy, College of Veterinary Medicine and Institute of Veterinary Science, Kangwon National University, Chuncheon, 24341 South Korea; 2 Department of Anatomy and Cell Biology, College of Veterinary Medicine, and Research Institute for Veterinary Science, Seoul National University, Seoul, 08826 South Korea; 3 Department of Biomedical Sciences, and Research Institute for Bioscience and Biotechnology, Hallym University, Chuncheon, 24252 South Korea; 4 Department of Biochemistry and Molecular Biology, Research Institute of Oral Sciences, College of Dentistry, Gangneung-Wonju National University, Gangneung, 25457 South Korea

**Keywords:** Fto, Obesity, Aging, Hippocampus, Mice

## Abstract

Fat-mass and obesity-associated protein (Fto) is highly expressed in the brain including, the hippocampus, and its expression is significantly decreased in the brain of Alzheimer’s disease patients. In the present study, we measured Fto immunoreactivity and protein levels in the hippocampus of obese and aged mice, which were induced by high-fat diet for 12 weeks and D-galactose treatment for 10 weeks, respectively. The obesity and aging phenotypes were assessed by physiological parameters and Morris water maze test, respectively. High fat diet fed mice showed significant increases in body weight and blood glucose levels compared to that in the control or D-galactose-induced aged mice. In addition, treatment with D-galactose significantly decreased the spatial memory. Fto immunoreactivity in the control group was mainly detected in the pyramidal cells of the CA1 and CA3 regions and in the granule cells of the dentate gyrus. In the hippocampus of high-fat diet-fed mice, Fto immunoreactive structures were similarly found in the hippocampus compared to that in the control group, but Fto immunoreactivity in high-fat diet-fed mice was also found in the stratum oriens and radiatum of the CA1 and CA3 regions and the polymorphic layer of the dentate gyrus. In the hippocampus of D-galactose-induced aged mice, fewer Fto immunoreactive structures were detected in the granule cell layer of the dentate gyrus compared to the control group. Fto mRNA and protein levels based on quantitative real-time polymerase chain reaction and western blot assays were slightly increased in the hippocampus of high-fat diet-fed mice compared to that in control mice. In addition, Fto mRNA and protein levels were significantly decreased in the aged hippocampus compared to that in the control group. Fto protein levels are susceptible to the aging process, but not in the hippocampus of high-fat diet-induced obesity. The reduction of Fto in aged mice may be associated with reduced memory impairment in mice.

## Introduction

Overweight/obesity and aging are the most threatening factors in human beings and about one fourth of the population in the world will be affected by overweight or obesity in 2030 [1]. In addition, the overall mean age will be 44 by 2020 compared to 40 in 2010 in the more developed countries classified by the United Nations. The fat-mass and obesity-associated (*Fto*) gene is located on chromosome 16 (16q12.2) and controls energy balance, food intake, and lipid metabolism in the body [2]. Fto is strongly associated with obesity [3] and aging [4]. Overexpression of Fto leads to increases in body and fat mass [5] and modulates the mitotic clonal expansion of adipose tissue in obese mice [6]. In contrast, mice with knockout or missense mutations of Fto have a lean phenotype with or without growth retardation [7, 8].

Fto mRNA and protein levels are highly abundant in various brain regions [9–11] including the hippocampus [12]. In patients with Alzheimer’s disease, Fto expression is decreased in the cortex and the amygdala [4] compared to tissue from control patients. Deficiency of Fto affects the brain size and distinct brain structures in mice [13]. In addition, knockout of Fto significantly reduces the expression of brain-derived neurotrophic factor (BDNF) in the hippocampus [13], delays the fear memory formation [14], and decreases learning and memory performance [13]. Fto also affects hippocampal neurogenesis and synaptic plasticity [13–15]. Phenotypes of Fto are similar to obesity- or age-induced impairment in hippocampal neurogenesis and reductions in BDNF expression. In previous studies, we found that obese and aged animals induced by high-fat diet (HFD) feeding for 4 or 12 weeks and D-galactose treatment for 7 weeks show less proliferating cells and differentiated neuroblasts in the dentate gyrus compared to mice in control group [16–19]. In addition, obese and aged animals show less expression of BDNF in the hippocampus compared to mice in the control group [17, 18].

However, only some studies were conducted to elucidate regional changes in Fto in the hypothalamus [11, 20, 21], not in the hippocampus, of obese and aged mice. In the present study, therefore, we investigated the localization of Fto immunoreactivity, protein and mRNA levels in the hippocampus of obese and aged mice to elucidate the role of Fto in the hippocampus of these animals.

## Materials and methods

### Experimental animals

Male C57BL/6 J mice (7 weeks old) were purchased from Jackson Laboratory Co. Ltd. (Bar Harbor, ME, USA). Five mice were housed per cage in a conventional area under standard conditions at ambient temperature (22 °C ± 2 °C) and humidity (60 ± 5%), with a 12/12 h light/dark cycle and ad libitum access to food and water. The handling and care of the animals conformed to guidelines compliant with current international laws and policies (NIH Guide for the Care and Use of Laboratory Animals, NIH Publication No. 85–23, 1985, revised 2011). The experimental protocol for using animals was approved by the Institutional Animal Care and Use Committee of Kangwon National University (KW-170613-2). All experiments were conducted with an effort to minimize the number of animals used and the suffering caused by the procedures employed in the present study [16, 18, 19].

### Induction of obesity and aging

Forty-eight mice were divided into control, obese, and aged groups. To induce obesity by HFD feeding, mice at 5 weeks of age were adapted to a chow diet for 1 week. Then the animals were fed commercially available HFD (D12492i; 60% fat, 20% protein, 20% carbohydrates, Research Diets) for 12 weeks. For the D-galactose-induced aged model, mice at 8 weeks of age received subcutaneous injections of D-galactose (Sigma-Aldrich, St. Louis, MO, USA) every day for 10 weeks. This protocol was chosen because the physiological obesity and aging phenotypes and hippocampal morphology of mice subjected to this protocol are well established [16–18].

### Phenotyping of obesity and aging

To confirm the diet-induced obesity, body weight in all mice (*n* = 16 in each group) was monitored in every weeks and blood glucose levels were measured at sacrificing time. Spatial memory impairments in control and D-galactose-treated groups were assessed by Morris water maze test described in the previous study [22]. Briefly, swimming speed and distance as well as time consumed to find the hidden platform was recorded for 4 consecutive days via a visual tracking system (Noldus Information Technology, Wageningen, The Netherlands). In addition, probe test was performed on the next day with removal of platform to elucidate the spatial memory judged from time spent in the target quadrant and in the three non-target quadrants (right, left, and opposite quadrants).

### Tissue processing and immunohistochemistry

At 18 weeks of age, mice (*n* = 5 in each group) were anesthetized with a mixture of alfaxalone (Alfaxan, 75 mg/kg; Careside, Seongnam, South Korea) and xylazine (10 mg/kg; Bayer Korea, Seoul, South Korea) and were perfused transcardially as described previously [23]. Paraffin brain section of 3 μm thickness were obtained using a microtome (Leica, Wetzlar, Germany) and 5 tissue sections, 90 μm apart from each other, were selected from an area between 1.82 and 2.30 mm posterior to the bregma based on a mouse brain atlas [24]. Immunohistochemical staining was conducted as described previously. Briefly, the slide-attached sections were put in 100 mL jars filled with citrate buffer (pH 6.0) and heated in a 2100-retriever (Prestige medical, Lancashire, UK) for antigen retrieval. The sections were successively incubated with rabbit anti-Fto antibody (diluted 1:500, ThermoFisher Scientific, Waltham, MA, USA) for 24 h at 25 °C, biotinylated goat anti-rabbit IgG antibody for 2 h at 25 °C, and a streptavidin-peroxidase complex (1:200; Vector Laboratories, Burlingame, CA, USA) for 2 h at 25 °C. All sections were visualized by reaction with 3,3′-diaminobenzidine tetrachloride (Sigma) in a 0.1 M Tris-HCl buffer (pH 7.2) solution and were dehydrated and mounted in Canada balsam (Kanto Chemical, Tokyo, Japan).

### Western blot analysis

To quantify changes in Fto levels in the whole hippocampus, 6 animals were euthanized with a mixture of 75 mg/kg alfaxalone and 10 mg/kg xylazine 2 h after the last D-galactose treatment. Left and right hippocampal tissues were acquired from the brain and used for western blot analysis as described in a previous study [25]. Briefly, the tissue samples from two animals were pooled in a single vial and homogenized in 50 mM phosphate buffered saline (pH 7.4) for western blot analysis. Aliquots containing 20 μg of total protein were heat denatured in the loading buffer and were then loaded onto a polyacrylamide gel. The proteins were then transferred onto nitrocellulose membranes (Pall Crop, East Hills, NY, USA) and sequentially incubated with rabbit anti-Fto antibody (diluted 1:1000) at 25 °C, a peroxidase-conjugated anti-rabbit IgG antibody (1:200; Vector), and an enhanced luminol-based chemiluminescent kit (Pierce Chemical). Data were normalized to the β-actin level in each lane.

### RT-qPCR experiments

For quantitative real-time polymerase chain reaction (RT-qPCR), animals (*n* = 5 in each group) was were euthanized with a mixture of 75 mg/kg alfaxalone and 10 mg/kg xylazine 2 h after the last D-galactose treatment. Brain was quickly removed from the skull and cut with vibratome (Leica). Hippocampal CA1 region, CA3 region, and dentate gyrus was dissected under stereoscope and tissue samples were immediately immersed in RNAlater solution (Qiagen) and RNA extraction was performed using a total RNA isolation kit (Macherney-Nagel). RT-qPCR was performed as described in the previous studies [12, 26] and the primers used were as follows: 5′-GGACATCGAGACACCAGGAT− 3′ (forward) and 5′-AGGTGCCTGTTGAGCACTCT-3′ (reverse) for FTO (accession number: NM_011936) and 5′-GCACCACACCTTCTA CAATG-3′ (forward) and 5′-TGCTTGCTGATCCACATCTG-3′ (reverse) for β-actin.

### Data analysis

Immunohistochemical data for Fto were analyzed in the hippocampal CA1 and CA3 regions and the dentate gyrus using ImageJ software v. 1.52 (National Institutes of Health, Bethesda, MD, USA) as previously described [25]. Briefly, images were acquired with a BX51 light microscope (Olympus, Tokyo, Japan) equipped with a digital camera (DP72, Olympus). The images were calibrated into an array of 512 × 512 pixels. Each pixel’s resolution was 256 Gy levels. The intensity of Fto immunoreactivity was evaluated by relative optical density (ROD), which was obtained after transformation of the mean gray level using the formula: ROD = log_10_ (256/mean grayscale level). ROD of background staining was determined in unlabeled portions of the sections using Photoshop CC 2018 software (Adobe Systems Inc., San Jose, CA, USA). This value was subtracted to correct for nonspecific staining, using ImageJ software v. 1.52 (NIH). Data are expressed as a percentage of the sham-operated group values (set to 100%).

### Statistical analysis

As previously described [25], data are shown as mean ± standard deviation. Differences among the means were statistically analyzed by one-way analysis of variance followed by Bonferroni’s post-hoc test, using GraphPad Prism 5.01 software (GraphPad Software, Inc., La Jolla, CA, USA). Statistical significance was considered at *p* < 0.05.

## Results

### Confirmation of obesity and aging in mice

**Fig. 1:**
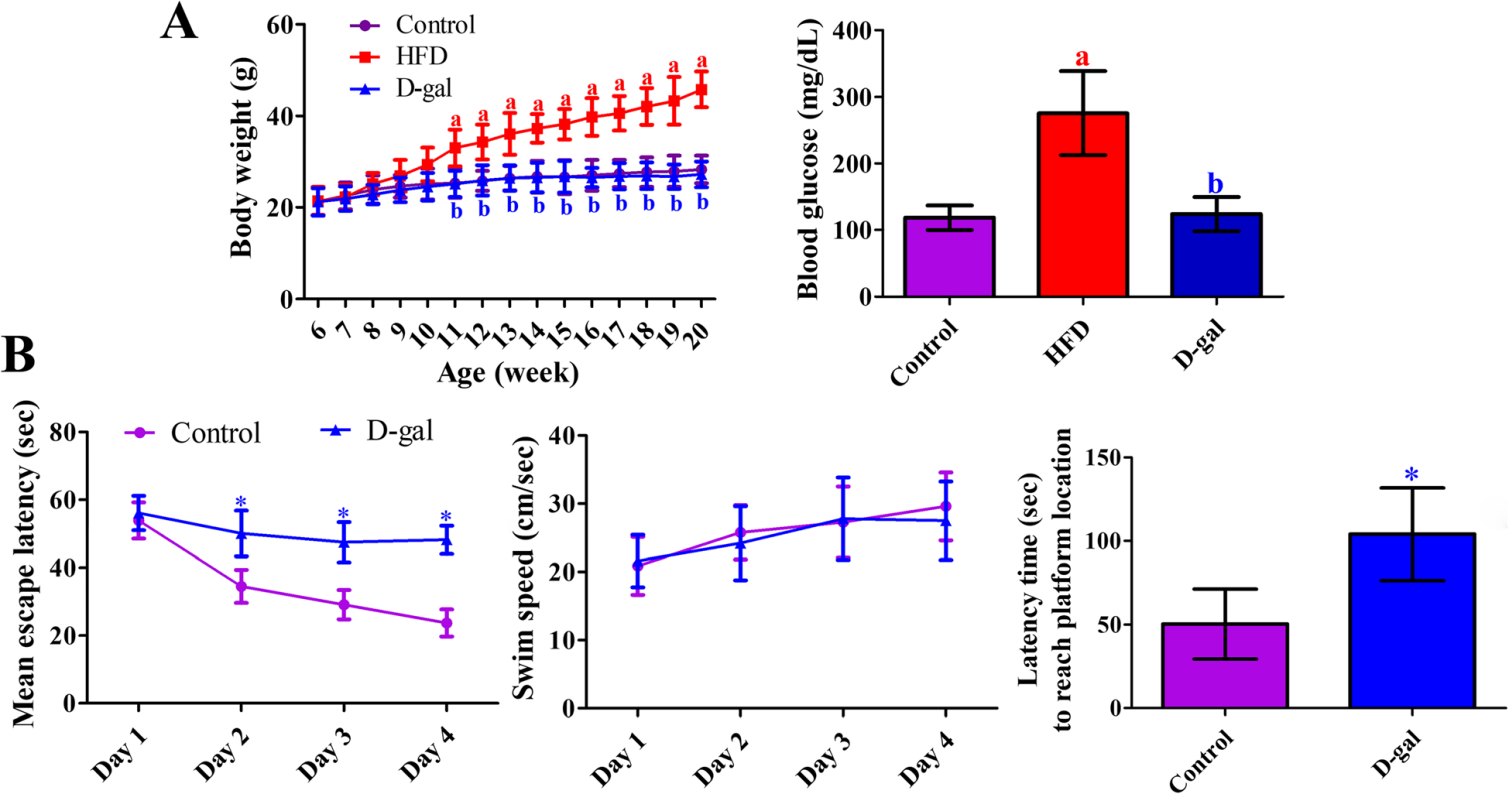
Physiological parameters (**a**) such as body weight and blood glucose levels in the control, high-fat diet-fed obese (HFD), and D-galactose-treated aged (D-gal) mice. In addition, spatial memory assessed by Morris water maze test (**b**) in the control and D-gal mice (*n* = 5 in each group. The data are analyzed by Student t-test or one-way analysis of variance followed by Bonferroni’s post-hoc test. ^a^*p* < 0.05, significantly different from the control group; ^b^*p* < 0.05, significantly different from the HFD group). The bars indicate the mean values ± standard deviation

In all groups, body weight was increased with age and the was significantly increased from 12 weeks of age in HFD-fed obese group compared to that in the control or D-galactose-induced aged group. In addition, mice in obese group at 20 weeks of age showed higher blood glucose levels compared to other groups (Fig. 1a).

Morris water maze test revealed that mean escape latency was significantly longer in the aged group compared to that in the control group at day 2, 3, and 4 of testing trial. However, there were no significant differences on the swimming speed. In the probe trial, mice in aged group spent longer time to reach platform location and showed significant decreases in number of crossings in the target quadrant (Fig. 1b).

### Changes of Fto immunoreactivity and mRNA levels in CA1 region

**Fig. 2:**
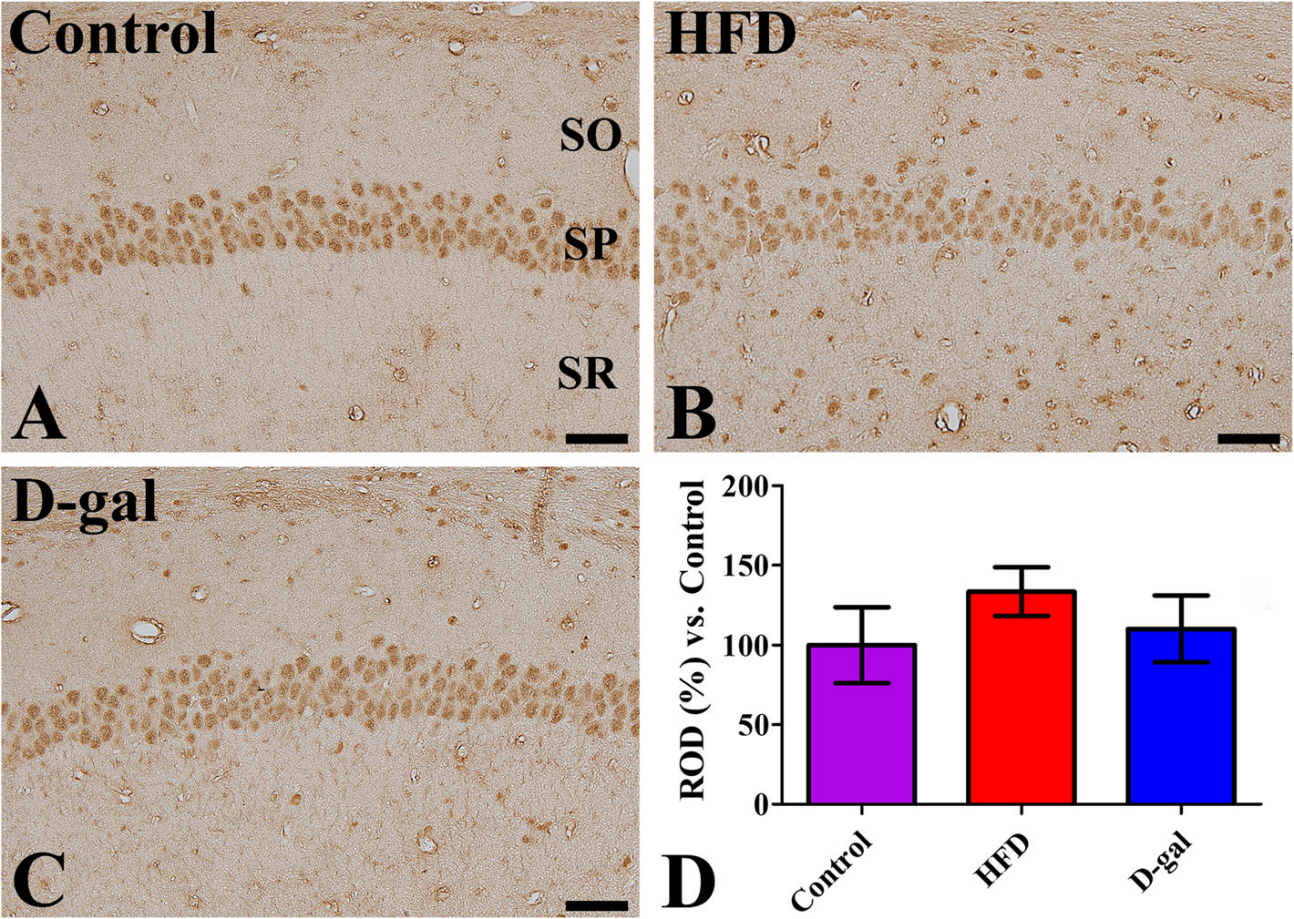
Microphotographs of Fto immunoreactivity in the hippocampal CA1 region in control (**a**), high-fat diet-fed obese (**b**, HFD), and D-galactose-treated aged (**c**, D-gal) mice. Note that Fto immunoreactivity in all groups is mainly found in the stratum pyramidale (SP), while Fto immunoreactive cells are also abundantly detected in the stratum oriens (SO) and the stratum radiatum (SR) of HFD-fed mice. Scale bar = 50 μm. **d** Relative optical densities (ROD) are expressed as a percentage of the value of Fto immunoreactivity in the control hippocampal CA1 region per section (*n* = 5 in each group. The data are analyzed by one-way analysis of variance followed by Bonferroni’s post-hoc test. There are no significant differences in ROD of Fto between groups. The bars indicate the mean values ± standard deviation

In the control group, Fto immunoreactivity was mainly observed in the pyramidal neurons of the CA1 region although a few Fto immunoreactive cells were also found in the strata oriens and radiatum. In addition, Fto immunoreactive neuropil was detected in the stratum radiatum (Fig. 2a). In obese mice, Fto immunoreactivity was abundantly detected in the pyramidal neurons, but Fto immunoreactive structures were also widely found in the strata oriens and radiatum (Fig. 2b) and Fto immunoreactivity was increased compared to mice in the control group (Fig. 2d). In aged mice, Fto immunoreactive nuclei were found in the stratum pyramidale and Fto immunoreactive neuropil was observed in the stratum radiatum (Fig. 2c). In this group, Fto immunoreactivity in the CA1 region was similar to that in control mice (Fig. 2d).

### Changes of Fto immunoreactivity in CA3 region

**Fig. 3:**
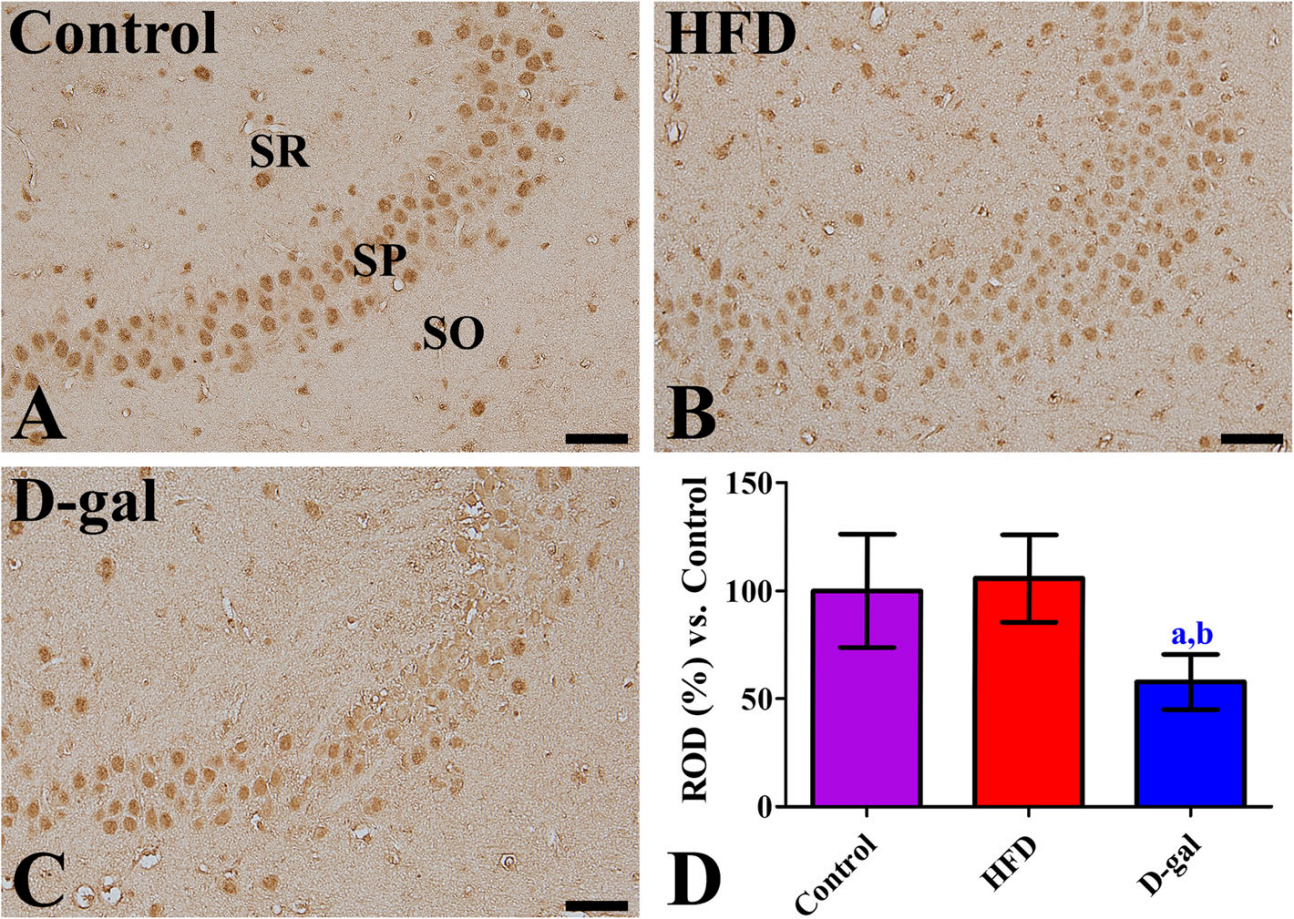
Microphotographs of Fto immunoreactivity in the hippocampal CA3 region in control (**a**), high-fat diet-fed obese (**b**, HFD), and D-galactose-treated aged (**c**, D-gal) mice. SO, stratum oriens; SP, stratum pyramidale; SR, stratum radiatum. Scale bar = 50 μm. **d** Relative optical densities (ROD) are expressed as a percentage of the value of Fto immunoreactivity in the control hippocampal CA3 region per section (*n* = 5 in each group. The data are analyzed by one-way analysis of variance followed by Bonferroni’s post-hoc test. ^a^*p* < 0.05, significantly different from the control group; ^b^*p* < 0.05, significantly different from the HFD group). The bars indicate the mean values ± standard deviation

In control mice, strong Fto immunoreactive nuclei were found in the stratum pyramidale of CA3 region and some Fto immunoreactive cells were also detected in the strata oriens and radiatum of CA3 region (Fig. 3a). In obese mice, Fto immunoreactive cells were abundantly found in the strata pyramidale and radiatum (Fig. 3b). In this group, Fto immunoreactivity was similarly observed in the hippocampal CA3 region compared to that in the control mice (Fig. 3d). In aged mice, Fto immunoreactive nuclei were mainly found in the stratum pyramidale (Fig. 3c), but Fto immunoreactivity was significantly decreased compared to that in the control mice (Fig. 3d).

### Changes of Fto immunoreactivity in dentate gyrus

**Fig. 4:**
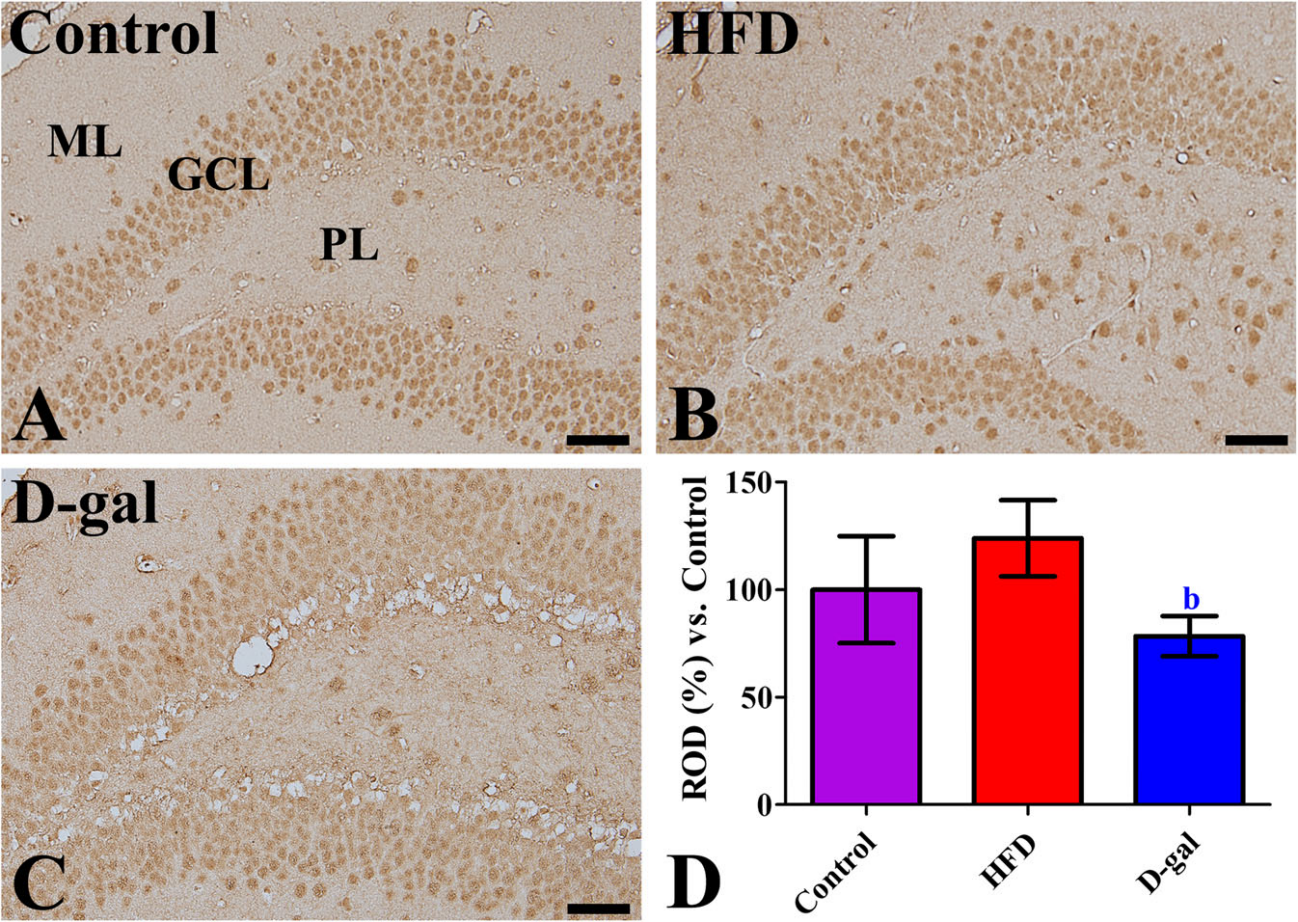
Microphotographs of Fto immunoreactivity in the dentate gyrus in control (**a**), high-fat diet-fed obese (**b**, HFD), and D-galactose-treated aged (**c**, D-gal) mice. Note that Fto immunoreactivity is mainly observed in the granule cell layer (GCL) in all groups, while Fto immunoreactivity in the HFD group is also detected in the polymorphic layer (PL). Scale bar = 50 μm. **d** Relative optical densities (ROD) are expressed as a percentage of the value of Fto immunoreactivity in the control in the hippocampal dentate gyrus per section (*n* = 5 in each group. The data are analyzed by one-way analysis of variance followed by Bonferroni’s post-hoc test. ^b^*p* < 0.05, significantly different from the HFD group). mean values ± standard deviation

In control mice, Fto immunoreactive cells were mainly observed in the granule cell layer of dentate gyrus. A few Fto immunoreactive cells were also found in the polymorphic layer (Fig. 4a). In obese mice, Fto immunoreactive cells were abundantly detected in the granule cell layer as well as in the polymorphic layer (Fig. 4b). In this group, Fto immunoreactivity was slightly increased in the dentate gyrus compared to that in control mice (Fig. 4d). In aged mice, Fto immunoreactivity was mainly observed in the granule cell layer (Fig. 4c), but Fto immunoreactivity was significantly decreased in the dentate gyrus compared to that in obese mice, not in the control mice (Fig. 4d).

### Changes of Fto mRNA and protein levels

**Fig. 5:**
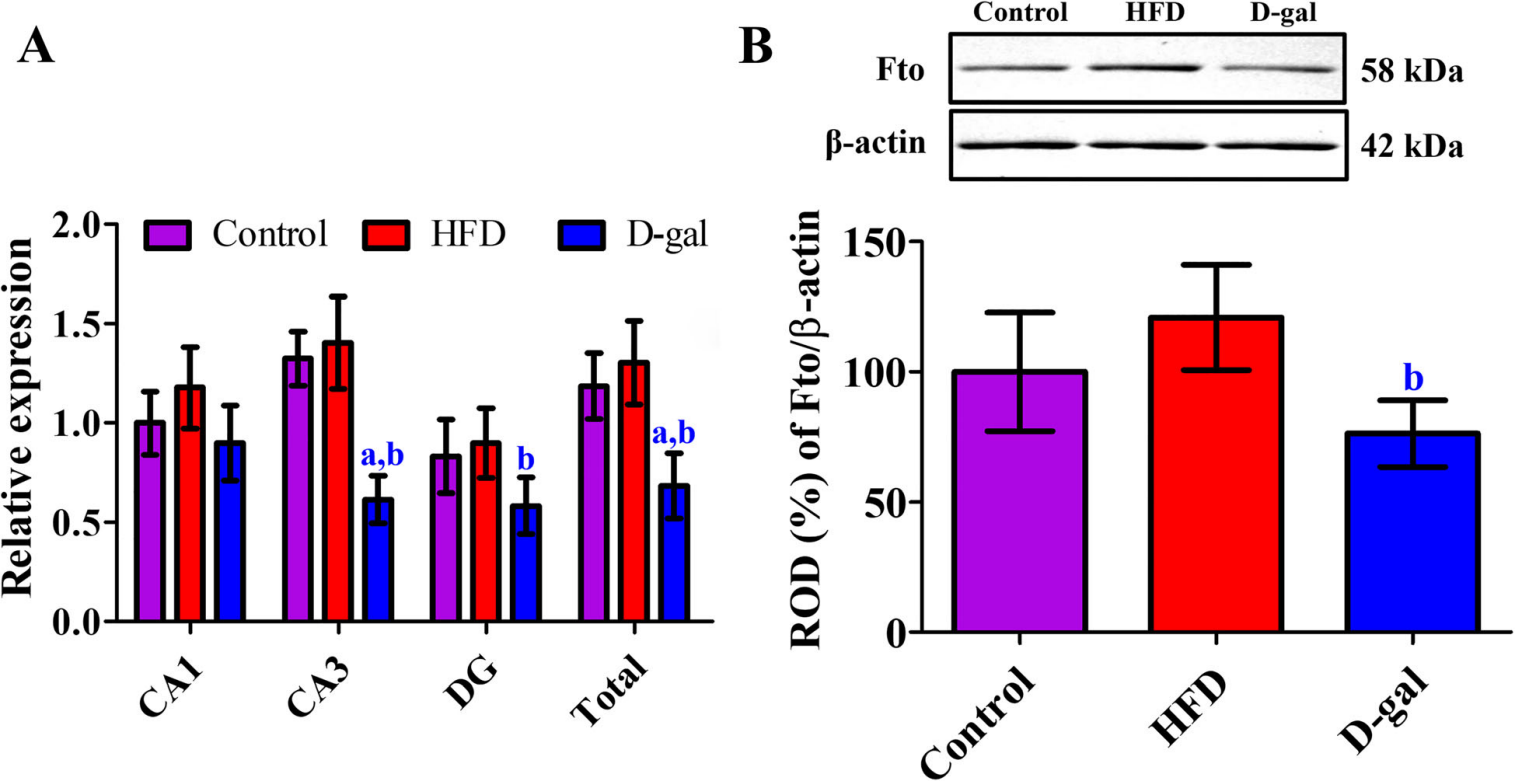
Quantitative real-time polymerase chain reaction (RT-qPCR, **a**) and western blot (**b**) analysis of Fto in the hippocampi of control, high-fat diet-fed obese (HFD), and D-galactose-treated aged (D-gal) mice. Values from RT-qPCR and western blot analysis are normalized as a ratio of the Fto and β-actin immunoblot bands in the control CA1 region and control group, respectively (*n* = 5 or 6 in each group, the data are analyzed by one-way analysis of variance followed by Bonferroni’s post-hoc test. ^a^*p* < 0.05, significantly different from the control group; ^b^*p* < 0.05, significantly different from the HFD group). The bars indicate mean values ± standard deviation

Fto mRNA levels did not show any significant changes in the hippocampal CA1 region of obese or aged mice compared to that in the control group. In contrast, Fto mRNA levels in the CA3 region and dentate gyrus were higher in the obese mice than in the control mice, but Fto mRNA levels were significantly decreased in hippocampal CA3 region of aged mice compared to that in the control mice. In total, Fto mRNA in whole hippocampal homogenates was significantly decreased in the aged mice compared to that in the control or obese mice (Fig. 5a).

Fto protein levels were slightly increased in the hippocampus of obese mice compared to control mice. In contrast, aged mice showed prominently lower levels of Fto in the hippocampal homogenates and there was significant reduction in in Fto protein levels compared to obese mice (Fig. 5).

## Discussion

Several lines of evidence demonstrate that Fto is abundantly expressed in the brain [10, 12]. Fto in the hypothalamus is related to regulating energy and food intake [11, 20, 21, 27]. In contrast, Fto in the prefrontal cortex is involved in memory formation [14, 15]. In the present study, we observed Fto immunoreactivity in the nuclei of pyramidal cells in the CA1 and CA3 regions as well as in granule cells in the dentate gyrus. This result is consistent with a previous study that showed that Fto expression is found in the nuclei and dendrites of hippocampal CA1 neurons in mice [28].

HFD results in impairments in memory and synaptic plasticity in the elderly [29, 30] and decreases in cognitive performance in children [31], showing similar phenotypes as in aging. In addition, HFD and aging processes reduce the hippocampal neurogenesis in the subgranular zone of the dentate gyrus, while Fto plays an important role in adult neurogenesis and memory formation [13]. In the present study, we observed changes in Fto immunoreactivity in the hippocampus of HFD-fed mice and D-galactose-induced aged mice. In the present study, we confirmed the HFD-induced obesity and D-galactose-induced aging phenotypes using body physiological parameters (body weight and blood glucose level) and behavioral test. We selected C57BL/6 strain because this strain exhibits the preference for protein and fat without any abnormalities in insulin, leptin and triglyceride levels when compared to other strains [32]. In addition, C57BL/6 strain has good learning ability in the Morris water maze test [33] and memory impairments can be distinguished in the mice using Morris water maze test. Control, HFD-fed obese, and D-galactose-treated aged mice showed strong Fto immunoreactivity in the stratum pyramidale of the hippocampal CA1 and CA3 regions as well as in the granule cell layer of the dentate gyrus. In obese mice, Fto immunoreactivity was also found in the strata oriens and radiatum of the CA1 and CA3 regions as well as in the polymorphic layer of the dentate gyrus. Fto expression is increased 2.5-fold in the arcuate nucleus by feeding of 45% HFD for 10 weeks [20] and that Fto decreased in the arcuate nucleus 48 h after fasting [10]. In contrast, decreasing Fto in the hippocampus by HSV-CRISPR/Cas9 or shRNA technology specifically enhanced contextual fear memory [28]. However, in the present study, we could not observe any significant changes of Fto immunoreactivity, protein, and mRNA levels in hippocampal subregions. This result suggests that HFD-induced obesity is less vulnerable to Fto in the hippocampus compared to arcuate nucleus.

In aged mice, Fto immunoreactivity was decreased in the hippocampal CA3 region and dentate gyrus compared to control mice. There have been conflicting data on Fto expression in the brain at various conditions. Fto expression is decreased in the rat hippocampus and cortex in an animal model of Parkinson’s disease [34] and in the cerebral cortex of mice exposed to arsenite [35]. Fto knockout mice show low expression of BDNF and disturbed ratio of proBDNF and mature BDNF in the hippocampus [13] and have impaired working memory [36], while Fto depletion slows down the cognitive impairments in mouse models of Alzheimer’s disease [37]. In the present study, we observed the reduction of Fto immunoreactivity, protein, and mRNA levels in the dentate gyrus of aged mice.

## Conclusion

Fto expression is decreased in the pyramidal cells of CA3 region and granule cell layer of the dentate gyrus in aged mice, while Fto expression is relative resistant to HFD-induced obesity although both obesity and aging affect hippocampal function. The reduced expression of Fto may be associated with impairments of hippocampal functions in aging.

## Data Availability

The datasets generated and/or analyzed during the current study are available from the corresponding author on reasonable request.

## Abbreviations

BDNF: Brain-derived neurotrophic factor
Fto: Fat-mass and obesity-associated
HFD: High-fat diet
ROD: Relative optical density
RT-qPCR: Quantitative real-time polymerase chain reaction
